# Simvastatin induces adverse effects on proliferation and mineralization of human primary osteoblasts

**DOI:** 10.1186/s13005-020-00232-4

**Published:** 2020-08-20

**Authors:** Martin Mariano Isabelo Sabandal, Edgar Schäfer, Jonathan Aed, Susanne Jung, Johannes Kleinheinz, Sonja Sielker

**Affiliations:** 1 Central Interdisciplinary Ambulance in the School of Dentistry, University of Münster, Albert-Schweitzer-Campus 1, Gebäude W30, Waldeyerstr. 30, 48149 Münster, Germany; 2 Department of Cranio-Maxillofacial Surgery, University Hospital Münster, Münster, Germany

**Keywords:** Mineralization, Osteoblasts, Adverse effects, Simvastatin

## Abstract

**Background:**

Frequently statins were administered to reduce the LDL-concentration in circulating blood. Especially simvastatin (SV) is an often prescribed statin. Pleiotropic effects of these drugs were reported. Thus, the aim of this study was to evaluate effects of SV on osteoblastic mineralization.

**Methods:**

After informed consent primary osteoblasts were collected from tissue surplus after treatment of 14 individuals in the Department of Cranio-Maxillofacial Surgery, University Hospital Münster. The cells were passaged according to established protocols. Viability, mineralization capability and osteoblastic marker (alkaline phosphatase) were determined at day 9, 13 and 16 after adding various SV concentrations (0.05 μM, 0.1 μM, 0.5 μM, 1.0 μM). Statistical analysis was performed using the Kruskal-Wallis-test.

**Results:**

The cell cultures showed a time and dose-dependent significantly decreased viability (*p < 0.01*) and a significantly increased mineralization (*p < 0.01*) in a late mineralization stage after adding SV. The typical alteration of the alkaline phosphatase (ALP) levels during osteogenic differentiation was not recognizable.

**Conclusions:**

The pleiotropic effects found for different SV concentrations were possibly originated from other mineralization pathways beside the ALP induced one. Additionally, possible alterations of protein expression levels during mineralization and investigation of possible deviating application of SV in other treatment fields can be considered after gaining a deeper insight in the affected mechanisms.

## Background

The human bone is one of the highest mineralized tissues in human being. Although bone is highly mineralized a continuous remodelling of its structure depending on the physiological requirements is evident. The remodelling is balanced between osteoclasts which resorb bone and osteoblasts which build bone [1]. In between there are osteocytes which are the formerly osteoblasts but embedded in surrounding bone. The function of the osteocytes alters with increasing age [2]. Due to the function and other evident complex growing patterns many different alterations can influence the formation of bone [3].

Several regularly administered pharmaceutics are known to exert an impact on bone remodelling and bone homeostasis [4, 5]. Bisphosphonates and denosumab are frequently used to treat different types of cancer [4] and osteoporosis [4, 5]. Both medicaments exert direct effects on bone metabolism; bisphosphonates influence particularly the action of osteoclasts [5], while denosumab interacts with receptors which are necessary for physiological bone resorption [4]. Both medicaments are used regularly to influence the bone metabolism directly but there are also other pharmaceutics which show side effects influencing the bone. When administered during a short time only little undesired side effect has to be expected but a longer administration of such drugs can possibly show altering effects on the bone metabolism [6]. For instance antirheumatic agents like methotrexate cause a dose-dependent decrease of human osteoblast proliferation [6, 7], also antiepileptic drugs influence the bone metabolism by inducing the cytochrome P450 system [6].

The group of the statins is a common therapeutic agent to reduce the concentration of low density lipoproteins (LDL) in blood [8]. Statins were administered since the late 1980s [9]. Prior to the usage of statins the so called fibric acid derivatives (fibrates) were used to lower the blood concentration of triglycerides [10]. In contrast to the group of statins the fibrates show only little effect on the circulating LDL blood concentration [10]. But the statins reduce the circulating LDL-concentration in blood more effectively [10]. Simvastatin (SV) is a member of the group of statins and one of the first 3-hydroxy-3-methylglutaryl-CoA (HMG-CoA) reductase inhibitors effective in lowering the circulating LDL concentration in blood [9]. Up to now different types of statins are known and used depending on the diagnosis. The most frequent diagnosis prior to administration of statins is hypercholesterolaemia with associated increased risk of atherosclerosis and heart diseases including coronary heart disease and the risk of cardiac infarction [11]. Due to the dose-dependent higher risk of rhabdomyolysis the recommended daily maximum dose was reduced in 2013 by the American Heart Association (ACC/AHA) from 80 mg to 40 mg per day [12].

The target location of SV is a reversible inhibition of the HMG-CoA reductase within the cholesterol biosynthesis and mevalonate pathway. The inhibition leads to a reduced intracellular concentration of mevalonate which serves as a regulator of the HMG-CoA reductase. Additionally, the expression of LDL receptors is upregulated [11]. Due to the upregulation of the LDL receptor expression the cellular intake of LDL from the circulating blood is increased causing a lowering of the LDL within the circulating blood [11].

During the use of SV so called pleiotropic effects have been recognized. Only few studies investigated the possible pleiotropic effects upon human cells of other tissues than the targeted. The examined cells were adipose tissue cells [13, 14], myeloma cells [15], osteoblasts [16–18], alveolar fibroblasts [19] and bone marrow cells [20]. Pleiotropic effects have been reported like increased osteoblastic differentiation [20–22], promotion of the viability and proliferation of osteoblasts [16, 23] and improvement of the mineralization [17, 24–26].

Thus, the aim of the present study was to determine the influence of SV on mineralization capability and further effects on viability upon primary human osteoblastic cells.

## Methods

### Study design and ethics approval

The study evaluated adverse effects of SV upon 14 primary mandibular osteoblast cell cultures originated from different donors. Cell viability and effects on osteogenic markers (alkaline phosphatase (ALP)) and mineralisation capability were analyzed. The study was designed according to the “Declaration of Helsinki” and approved by the Ethics Committee of the Faculty of Medicine, University of Münster (#2016–444-f-S). Previous to the collection and cell isolation, a written informed consent was obtained from all donors.

The following exclusion criteria were determined:

- tumour on the head and/or neck
- systemic administration of statins prior to the study
- subjects younger than 18 years
- subjects with possible pregnancy

### Collection of the bone samples

All collecting procedures were performed anonymously and under sterile conditions. Human cancellous mandibular bone was collected from the tissue surplus as corticospongiosa bone fragments of patients treated by dysgnathia surgery, removals of osteosynthesis materials and osteotomies in order to remodel the bone contour at the Department of Craniofacial Surgery, University Hospital Münster.

### Isolation and cultivation of primary human osteoblast cell cultures

Isolation and culture techniques of primary human osteoblast cells were performed as described previously [27, 28]. For further cell culturing primary osteoblasts were cultivated in Dulbecco’s Modified Eagle Medium (DMEM) high glucose (DMEM high glucose, pyruvate; Gibco, Dreieich, Germany) supplemented with 12% bovine calf serum, 1% Amphotericin B [250 mg/mL], 1% Penicillin [10.000 U/mL] / Streptomycin [10.000 g/mL] and 1% glutamine [200 mM] (all Biochrom, Berlin, Germany). Cells were cultivated at 37 °C in a humidified atmosphere with 5% CO_2_. The culturing medium was replaced every 2 to 3 days and the cells were passaged after reaching 90% of confluence. For osteogenic differentiation 16 ng/mL dexamethasone (Fortecortin, Merck Pharma, Darmstadt, Germany) was added. For inducing mineralization ascorbic acid [1.4 mM] and ß-glycerophosphate [10 mM] was added additionally (all Sigma-Aldrich, Hamburg, Germany). SV was dissolved in ethanol_abs._ to a final stock solution of 6 mM and 1 mM stored at 4 °C (all Sigma-Aldrich). Various SV concentrations (0.05 μM, 0.1 μM, 0.5 μM and 1.0 μM) were prepared by diluting stock solution of SV with culturing medium. According to the dilution the final concentration of ethanol was 0.1% in the culturing medium with 1 μM SV.

Cells were seeded in a density of 5.000 cells/cm^2^ in 48-well plates (Greiner Bio-One, Frickenhausen, Germany) and allowed to adhere for 24 h before the application of SV which was freshly diluted in mineralization inducing culturing medium (0.05 μM, 0.1 μM, 0.5 μM, 1.0 μM). Freshly mixed culturing medium with SV was replaced every two to 3 days throughout the cell culture study. As control group, cells with osteogenic induction and without SV in culturing medium were used. Cell viability and osteogenic activity were analyzed at day 9, day 13 and day 16. Cell culture was performed with three replicates.

Primary human mandibular osteoblasts were characterized in two different ways [28].

- Positive immunohistochemical staining of osteonectin and collagen 1 in culture medium.
- Positive staining of osteocalcin (OC) and alizarin red S during mineralization in inducing culture medium.

As a negative control, cells in culture medium without SV and osteogenic induction were used.

### Determination of the nontoxic simvastatin concentration

Cells of two primary mandibular osteoblast cell cultures were seeded in a density of 10.000 cells/cm^2^ in 48-well plates (Greiner Bio-One, Frickenhausen, Germany). Cells were allowed to adhere for 24 h before SV freshly diluted in culturing medium (0.01 μM up to 20 μM) was added. As a control, cells without SV in culturing medium were used. Cell viability was analysed 24 h, 48 h, and 72 h after adding SV.

### Cell viability (MTT-assay)

Cell viability was estimated with an in-house MTT assay. The conversion of the yellow thiazolyl blue tetrazolium bromide (0.5 mg/mL) (Sigma-Aldrich) to the purple formazan dye by the cellular NAD(P) reflux was photometric measured at 570 nm wavelength. Cytotoxic effects were determined with the Pierce™ LDH Cytotoxicity Assay (ThermoFisher Scientific, Dreieich, Germany). All assays were performed according to manufacturing protocols and done in triplicate. μQuant™ reader (BioTek, Winooski, Vermont, USA) was used for colorimetric determination.

The qualitative analysis of cell viability was performed using fluorescein diacetate (FDA) / propidium iodide (PI) staining, FDA (Sigma Aldrich) stained viable cells green, and PI (Fluka, Sigma-Aldrich, Hamburg, Germany) stained nuclei of necrotic and apoptotic cells red.

### Osteogenic activity

For protein expression analysis and alkaline phosphatase activity, cells were lysed with the Pierce™ IP Lysis Buffer (ThermoFisher Scientific) according to manufacturer protocol and the supernatant was frozen at − 80 °C for subsequent assays. Protein quantification was performed with the Pierce™ BCA Protein Assay (ThermoFisher Scientific). Alkaline Phosphatase activity was detected with the Alkaline Phosphatase Assay Kit (abcam, Cambridge, UK). All assays were performed according to the manufacturer protocol. μQuant™ reader (BioTek) was used for colorimetric determination. For determination of mineralization capability a modified alizarin red S staining was used (Alizarin Red S Staining Quantification Assay, ScienCell, Carlsbad, USA) [29]. Cells were fixed with formaldehyde (4% in phosphate buffered saline) and stained with an alizarin red S solution (40 nM, pH 4.1). Staining results were documented by photography. For quantification, stained cells were lysed in 10% acetic acid, heated at 85 °C for 10 min, and centrifuged for 15 min with 20.000 x g. The supernatant was neutralized with a 10% ammonia solution. Resolved alizarin red S was measured at 405 nm wavelengths. μQuant™ reader (BioTek) was used for colorimetric determination.

### Statistical analysis

The statistical analysis was done using the statistical software SPSS version 25 (IBM, Ehningen, Germany). For each observation time the results of the samples were assigned to the concentration groups. Due to the non-normal distribution of the data the Kruskal-Wallis-test was used. Only the groups of the specific days were compared to each other. The level of significance was set at *p < 0.05*.

## Results

### Investigation of the toxicity limits of SV

**Fig. 1:**
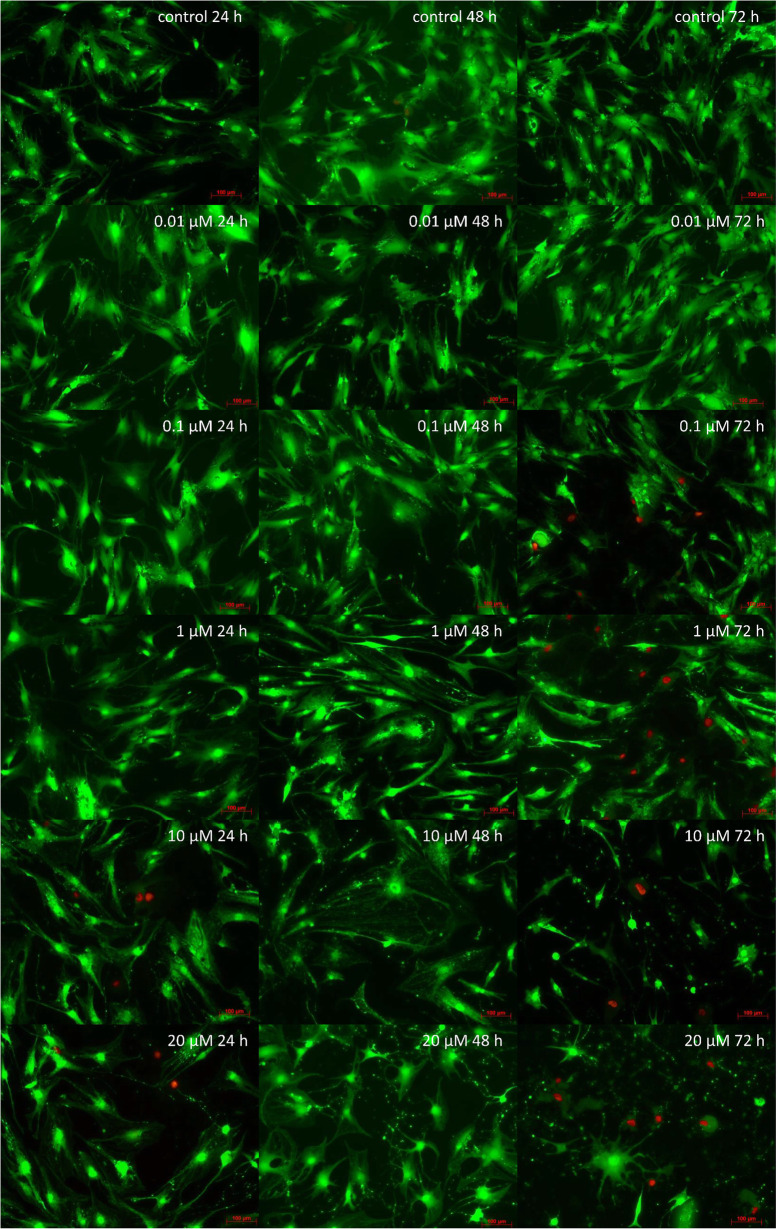
Live/dead staining: x-axis time, y-axis downwards ascending concentrations of added SV; viable cells green, necrotic and apoptotic cell nuclei red

Toxicity limits of SV were evaluated by live/dead staining after adding different concentrations of SV (0.01 μM, 0.1 μM, 1 μM, 10 μM and 20 μM) to the culture medium. The live/dead staining was performed at 24, 48 and 72 h. After 48 h SV concentrations of 10 μM and 20 μM showed marked cytotoxic effects. SV concentrations of 0.1 μM and 1 μM showed cytotoxic effects but the treated cells survived on a reduced level of viability (Fig. 1). According to the results the highest used SV concentration was 1 μM.

### Cell viability (MTT-assay)

**Fig. 2:**
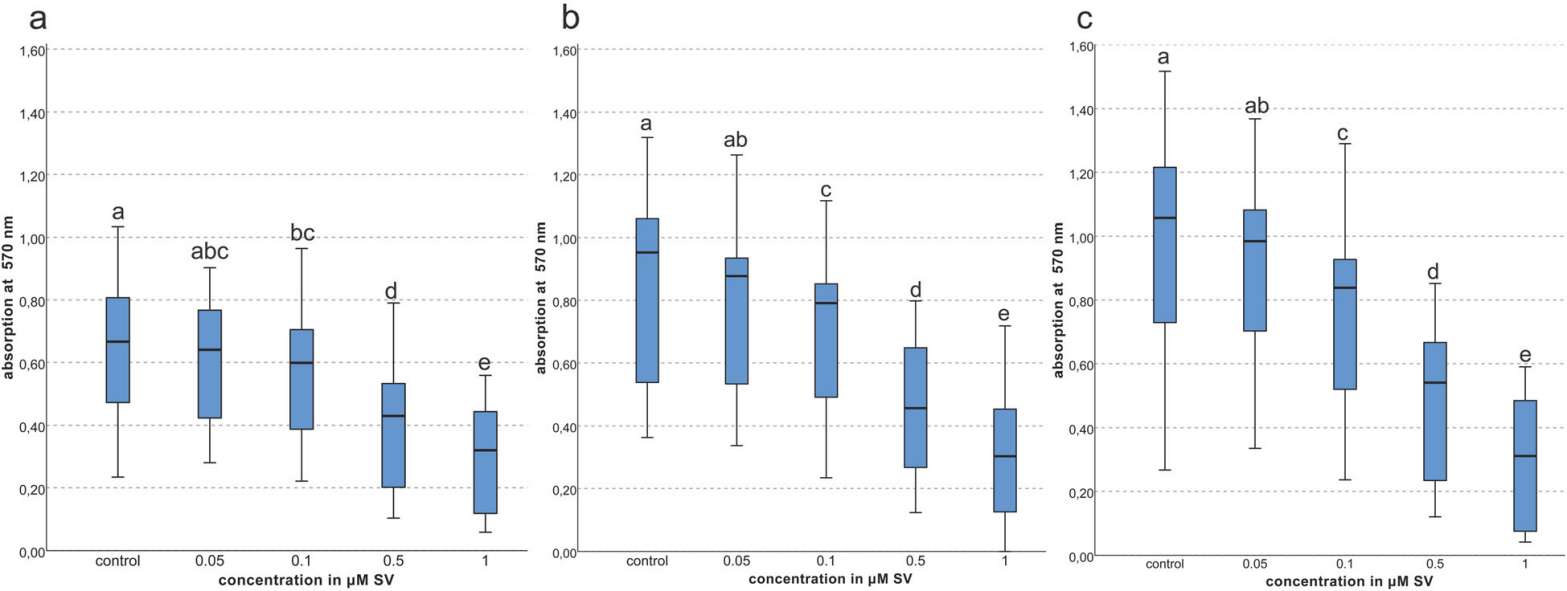
Viability given as photometric absorption at λ 570 nm of the MTT-assay on **a** = day 9, **b** = day13, **c** = day 16 (different letters indicate statistically significant differences at *p < 0.05*)

On day 9 concentrations of 0.5 and 1 μM SV showed a significant decrease (*p < 0.01*) of the viability of the primary osteoblast cultures compared to the groups of 0.05, 0.1 μM SV and the control group. Also the group of 0.1 μM SV showed significantly decreased cell viability (*p < 0.01*) compared to the control group. The decrease of the viability was inversely proportional to the SV concentration. Only little alterations of the cell viability in SV concentrations lower than 0.05 μM SV compared to the control group were visible (Fig. 2a).

On day 13 a concentration dependent decrease of the cell viability was evident. For SV concentrations of 0.1, 0.5 and 1 μM a significantly (*p < 0.01*) decreased cell viability compared to the control and the group with 0.05 μM SV was found. Compared with the cell viability on day 9 the results of the different groups with SV concentrations of 0.5 and 1 μM appeared nearly unaltered but a slight time dependent elevation of the control group and the groups with 0.05 and 0.1 μM SV were noticed (Fig. 2b).

On day 16 the viability of the cultures with 1 μM SV was nearly unaltered, whereas the values of the other groups slightly elevated compared to those of the corresponding groups on day 13. The groups with 0.1, 0.5 and 1 μM SV showed a significant (*p < 0.01*) decrease of the viability compared to the group with 0.05 μM SV and the control group (Fig. 2c). When comparing the values of the different times of investigation a time and concentration dependent decrease of the cell viability was obvious. SV concentrations of 1 μM exerted the most pronounced effect. In general, corresponding to the determination of the toxicity limits increasing concentrations of SV decreased cell viability (Fig. 2).

### Osteogenic marker (ALP-assay)

**Fig. 3:**
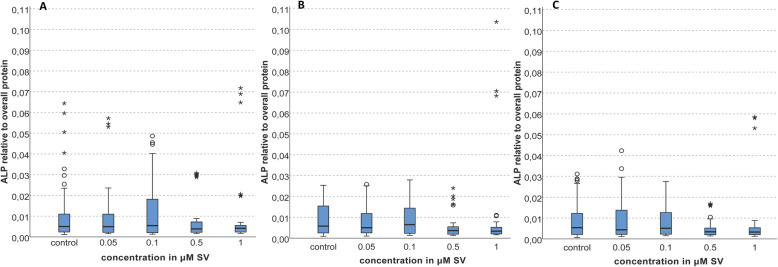
Conversion of ALP normalized to the overall protein; **a** = day 9 = **a**, **b** = day13 = **b**, **c** = day 16

The determination of the conversion of ALP is a typical marker of mineralizing cells. The statistical analysis of the ALP values revealed no significant alterations over time (*p > 0.05*). The results of day 9 showed a large distribution of the 50% quartile for the control group and the groups with 0.05 and 0.1 μM SV. The largest range of the 50% quartile was found in the group with 0.1 μM SV. Comparing the values of the groups with 0.5 and 1 μM SV with the other groups a smaller range was evident. The median value of all groups was nearly similar. Discordant values were distributed over all groups including the control group. The highest discordant values were found in the control group and the group with 1 μM SV (Fig. 3a).

On day 13 discordant values were evident only in the groups with 0.5 and 1 μM SV. The distribution of the 50% quartile of the control groups and the groups with 0.05 and 0.1 μM SV was larger than the 50% quartile of the groups with 0.5 and 1 μM. Compared to the results on day 9 the values of the control group and of the groups with 0.05 and 0.1 μM SV were slightly increased (Fig. 3b).

On day 16 the conversion of ALP slightly decreased in all groups including the control group compared to the values of the other days. Again discordant values were evident in all groups. The range of the 50% quartile was decreased in the control group and the group with 0.1 μM SV. The range in the groups with 0.5 and 1 μM SV was nearly unaltered over all days, the median values were nearly similar. In the groups with 0.5 and 1 μM the upper 25% quartile was increased compared to the results on days 9 and 13 (Fig. 3c).

### Mineralization (alizarin red S staining)

On days 9 and 13 the ranges of the 50% quartile and median values of all groups were nearly unaltered.

**Fig. 4:**
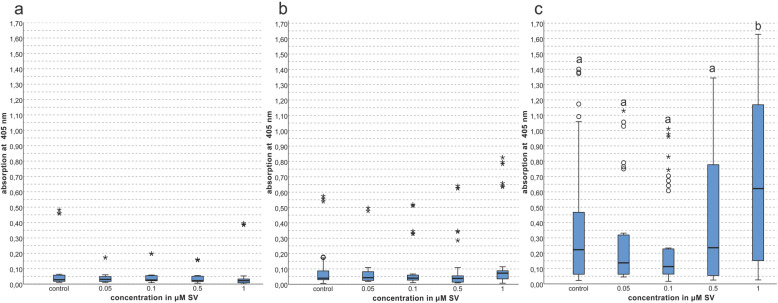
Mineralization given as photometric absorption at λ 405 nm of the alizarin red S staining; **a** = day 9 = **a**, **b** = day13 = **b**, **c** = day 16; (different letters indicate statistically significant differences at *p < 0.05*)

On day 16 a significantly increased (*p < 0.01*) mineralization in the group with 1 μM SV compared to all other groups and the control group was evident. The 50% quartile of the control group and of the groups with 0.5 and 1 μM SV were clearly increased compared to all other groups at day 16. The median value of the 1 μM group was about 2.5 fold elevated compared to the control group. Some discordant values were found especially with lower concentrations of 0.05 and 0.1 μM SV and in the control group but the range of these discordant values was below the maximum values of the groups with 0.5 and 1 μM SV (Fig. 4c).

## Discussion

The regular daily administration of SV ranges from 5 to 40 mg per day [12]. The corresponding systemic available concentration of SV ranges due to the biological availability of 5% from 0.05 to 5 μM. The SV concentrations used in the current study were chosen based on the performed cytotoxicity test and correlated with those (0.001 μM up to 10 μM) used in previous studies using human cells [13–18, 20, 29, 30]. Especially alterations of mineralization [14, 31], osteogenic markers like ALP, OC, receptor activator of nuclear factor kappa-Β ligand, bone morphogenetic protein 2, bone sialoprotein and osteoprotegerin [18, 20, 31] were investigated previously. Additionally, osteoblastic differentiation and proliferation were evaluated [16, 20, 31]. The studies investigating the above mentioned parameters were performed using human cells of different tissues like bone marrow cells and mesenchymal stem cells [20], periodontal ligament cells (PDL-cells) [17, 18, 31], cells from adipose tissues [13, 14], lung fibroblasts [19], immortalized multiple myeloma cells [15], osteoblast-like cell lines MG63 [30] and osteoblasts from bone chips [16].

In animal studies the influence on mineralization, osteogenic markers and viability of quiet equal concentrations of SV were investigated upon cells particularly of rodent origin [21–26, 32–39].

The present study investigated the influence of SV on osteoblasts from cancellous bone, which represents a more clinical approach to assess the influence of SV on mineralization in bone than using cell lines of rodent origin. Only one other study investigating the effects of SV on osteoblasts originated from bone is currently available [16]. The difference of the present study compared to previously published investigations is that higher SV concentrations of up to 1 μM SV were used (the highest SV concentration used on PDL-cells was 0.1 μM in previous studies [18, 31]) and a longer investigation period of up to 16 days was established.

ALP is a typical osteogenic marker [24, 40] during osteogenic differentiation [24]. During mineralization and osteogenic differentiation a higher amount of proteins is expressed [40]. Increased conversion of ALP corresponds to increased osteogenic activity [41]. The present study determined the conversion of ALP normalized to the total expressed protein according to the method described by Liu et al. and Zhao et al. [18, 31]. Increased ALP-activity under influence of SV as a possible sign of enhanced osteoblastic differentiation was found in bone marrow cells under the influence of 0.01 and 1 μM SV [20]. Cells of different tissues showed varying effects upon SV when comparing alveolar osteoblasts with PDL-cells [18]. The increase of the ALP conversion seems to be time and dose-dependent [31].

The present study revealed no significant alteration of the conversion of ALP for all SV concentration while Liu et al. found a significant increase of ALP within 24 and 48 h after exposition comparing the group with 0.001 μM SV to the control group, but after 72 h the same group showed a marked decrease of ALP [18]. Other studies showed increased ALP levels after exposition [18, 20, 23, 24, 31, 39] and higher concentrations than 0.1 μM SV caused no further increase of ALP [18, 31]. The comparison of these previous findings with those of the present study are limited as different observation periods were established (up to 16 days in the present study versus up to 72 h in previous investigations [18]. Another study reported a significant increase of ALP in the groups with 0.01 and 0.1 μM SV compared to the control group on days 7 and 14 [31]. This observation is in contradiction with the current findings as nearly no alterations of the conversion of ALP using low concentrations of 0.05 and 0.1 μM SV compared to the control group were found. The 50% quartile and the absolute values of the groups with 1 and 0.5 μM SV were slightly reduced at every investigation time compared to the control group (Fig. 3).

The present investigation of the viability after exposition to SV was performed according to the methodology of other studies, but observation times were only up to 6 days in these previous studies [13, 16, 31]. Thus, a direct comparison between the current and the previous findings is limited due to the different observation times. Proliferation of PDL-cells was not affected by 0.01 and 0.1 μM SV during a 5 day period but during the same time concentrations of 1 and 10 μM SV significantly suppressed proliferation [13, 31]. This is in agreement with the results of the present study as significantly suppressed viability (*p < 0.01*) of the primary osteoblastic cells under the influence of SV was found for every investigation time except of the group with 0.05 μM SV, which showed no significant suppression (*p > 0.05*) compared to the control group (Fig. 2). Beside the influence of SV on the viability an impact of the used solvent ethanol_abs_ for SV preparation is thinkable but the final dilution of the used SV concentration of 1 μM corresponds to a dilution ratio of 1:1000 which is equal to 0.1%. Additionally, ethanol can be found physiologically in the metabolism of the osteoblastic cells. In disagreement to the present findings one study investigated the effects on primary osteoblasts from bone chips [16] and found a dose-dependent significant increase of proliferation and cell count with SV concentrations of 0.01, 0.05, 0.1 and 0.5 μM during the investigation period from 3 to 6 days [16]. Another study examined the influence of SV on alveolar osteoblasts for up to 5 days and concentrations of 0.1 and 0.01 μM SV exerted no influence after 5 days of treatment, while concentrations of 1 and 10 μM SV showed a significant suppression of cell proliferation at day 3 and 5 compared to the control group [31]. As shown above the results differ but in general a dose-dependent decrease of proliferation with increasing SV concentrations is evident [13].

A nearly unaltered conversion of ALP and a dose-dependent decrease of viability in cell cultures imply a reduced mineralization. The mineralization was determined according to a previously published protocol [27]. Alteration of mineralization was frequently investigated using alizarin red S staining to determine the mineralization capability of osteogenic cells in vitro [13, 20, 31]. The present results showed in the early stages of mineralization on days 9 and 13 no alteration of the mineralization but on day 16 a highly significant increase (*p < 0.01*) (Fig. 4c) when 1 μM SV was used. The present findings corroborate those of previous studies, which reported elevated mineralization in bone marrow cells with 1 μM SV [20] and in PDL cells with 0.01 and 1 μM SV [31]. The same effect of increased mineralization was found in cell culture studies using adipose tissues and SV loaded hydroxyapatite scaffolds on days 7 and 21 [14] and on PDL cells with 0.01 μM, 0.1 μM and 1 μM SV with the most pronounced effects when 0.1 μM SV was used [17].

In summary, the present findings revealed a SV induced reduction of the cell viability and an increase of mineralization as a pleiotropic effect which correlates with the findings of other investigations [13, 16, 31]. Although the proliferation of the osteoblastic cells was reduced, SV exerted an increased effect on osteoblastic mineralization capacity at a later stage of mineralization [14, 17, 20, 31] especially evident on day 16 in the present study. A time and dose-dependent cytotoxic effect of SV on human osteoblasts and likewise other cell types has been shown in the present and other studies [15, 20, 31]. The decreased viability of the cells (Fig. 2) and increased mineralization (Fig. 4) beside nearly unaltered levels of the typical osteogenic marker ALP [42, 43] imply a possible alternative pathway of mineralization beside the osteogenic effect of increased ALP conversion [42, 43]. Regularly, a reduced cell count originated by decreased viability (Fig. 2) is supposed to reduce expression of cell products but the present findings showed a nearly unaltered conversion rate of ALP relative to the total protein count. The increase of the ratio of ALP to the total protein related to the alteration of viability is possible but was not determined.

## Conclusions

SV caused a time and dose-dependent significant decrease of cell viability and a significant increase of mineralization in a late mineralization stage while the alkaline phosphatase turnover was nearly unaltered.

Pleiotropic effects of SV have been reported earlier, especially in animal studies. In particular, a favourable influence of simvastatin in the healing of bone defects, e.g. by apical periodontitis, may be conceivable. A corresponding influence of statins on the healing tendency was investigated by Alghofaily et al. [44]. The results of the study show a significantly improved healing of apical periodontitis upon systemic administered statins. Pleiotropic effects in human cells are known but a deeper insight in the explicit metabolic pathways especially of the mineralization pathway has to be established. Further investigations should evaluate possible effects of SV on different cell types and tissues. Additionally, possible alterations of protein expression levels during mineralization and investigation of possible application of SV in other treatment fields can be considered after gaining a deeper view in the cellular mechanism of SV.

## Data Availability

The datasets analyzed during the current study are available from the corresponding author on reasonable request.
